# Age-related alterations in vortex veins on indocyanine green angiography

**DOI:** 10.1007/s11357-024-01298-7

**Published:** 2024-08-15

**Authors:** Chen-Xi Cai, Shan-Shan Yu, Xiao-Mei Xiong, Bing-Qian Liu, Zhen-Qiang Lin, Qiang Wang, Jin-Li Cui, Ze-Hao Liu, Tao Li, Lin Lu, Ying Lin

**Affiliations:** 1 State Key Laboratory of Ophthalmology, Guangdong Provincial Key Laboratory of Ophthalmology and Visual Science, Clinic Department, Zhongshan Ophthalmic Center, Sun Yat-sen University, Tianhe District, No. 7, Jinsui Road, Guangzhou, 510060 China; 2 Capital Medical University Electric Teaching Hospital, State Grid Corporation of China Beijing Electric Power Hospital, Beijing, 100073 China

**Keywords:** Age, Vortex veins, Choroid, Indocyanine green angiography

## Abstract

To determine age-related alterations in vortex veins in healthy subjects. A total of 228 healthy subjects (aged 4 to 86 years) were recruited and divided into four groups (G1, <21 years; G2, 21–40 years; G3, 41–60 years; and G4, 61–86 years). The clinical characteristics of the participants were recorded, and parameters including the number of vortex vein roots (NVVR), the central vortex vein diameter (CVVD), the mean root area of the vortex vein (MRAVV), and the weighted mean of the thickest branch diameter (WMTBD) were obtained by marking the vortex veins on indocyanine green angiography (ICGA). The NVVR in the age group over 60 years old was significantly lower than that in other age groups (*P* < 0.05). The CVVD, MRAVV, and WMTBD of all age groups increased with increasing age (*P* < 0.05). The NVVR was unevenly distributed among the quadrants (*P* < 0.001). The proportions of type four vortex veins (complete systems including ampulla) and anastomotic branches of the vortex veins were significantly increased in elderly participants over 50 years of age (*P* < 0.05). Subfoveal choroidal thickness was significantly correlated with age, NVVR, CVVD and MRAVV (*P* < 0.05). This is the first study to reveal age-related alterations in vortex veins on ICGA in a healthy population. Aging may lead to partial vortex occlusion and residual vortex dilation. As age increases, anastomotic branches increasingly appear between the originally independent vortex veins. *Translational relevance:* Aging may lead to partial vortex occlusion and residual vortex dilation.

## Introduction

Vortex veins are the major drainage channels for the choroid and are associated with many choroidal diseases, including vortex vein varices, uveal effusion syndrome, pachychoroid disease, and spaceflight-associated neuro-ocular syndrome, as well as complications of scleral buckle surgery [1]. Pachychoroid spectrum diseases are associated with altered vortex vein hemodynamics caused by vortex venous congestion [2]. Hirahara et al. demonstrated that the choroid of the eyes with central serous chorioretinopathy (CSC) had significantly higher blood vessel density than healthy controls, including the macular and peripheral regions [3]. It has been observed that through indocyanine green angiography (ICGA), eyes with pachychoroid spectrum diseases were more likely to have vortex venous anastomoses than other healthy eyes and show retrograde pulsatile blood flow [4–6]. Enlargement of the ampulla of the vortex vein, a condition called varix, can be confirmed with ICGA imaging. Vortex varicose veins have been misdiagnosed as uveal tumors, and there have been many cases where the eye has been mistakenly removed [7]. Therefore, it is necessary to further understand the vortex veins.

The vortex veins are located in the middle layer of the eye wall and are therefore difficult to detect under direct vision. Currently, methods for detecting vortex veins include color fundus photography, fundus autofluorescence, spectral-domain optical coherence tomography with enhanced depth imaging (SD-OCT+EDI) and fluorescein angiography, but ICGA can provide the best assessment. With the assistance of ICGA, we can observe the shape and location of the vortex veins more clearly than with other techniques [1].

Aging is closely associated with cardiovascular and neurodegenerative diseases and may cause tissue hypoperfusion, with dramatic effects on neural tissues with high metabolic demands (i.e., the brain and retina) [8, 9]. Many choroidal diseases are most likely to occur in middle-aged and elderly people, so it is speculated that the blood vessels and fossa veins of the choroid must change with age. Yu et al. [10] reported that macular perfusion decreased with aging according to measurements obtained using optical coherence tomography angiography (OCTA). Our previous research [11] revealed that decreased retinal tissue perfusion (RTP) and volumetric vessel density (VVD) in the deep vascular plexus along with increased VVD in the superficial vascular plexus (SVP) might be a characteristic pattern of normal aging in the healthy population. Matsumoto et al. [4] revealed that in patients with pachychoroid spectrum diseases, those with pulsatile vortex venous flow were much older than those without it. Concerningly, the average age of onset of polypoidal choroidal vasculopathy (PCV) is 68.4 years, which is mostly concentrated from 50 years old to 70 years old [12]. This suggests that age may be related to choroidal blood flow.

The goal of the present study is to describe the various parameters of vortex veins, to propose a new quantitative assessment of vortex veins and to determine age-related alterations in vortex veins in healthy subjects.

## Material and methods

Ethical approval for this retrospective study was obtained from the Institutional Review Board of Zhongshan Ophthalmic Center (No. 2022KYPJ173). In total, 228 participants (mean age, 44.18 ± 18.82 years; range, 4–86 years) were recruited in this study, including 117 males and 111 females. We retrospectively collected ICGA images from January 2015 to January 2022 at Zhongshan Ophthalmology Center, Guangzhou, China. We included patients whose contralateral eye was diagnosed with unilateral disease and with no choroidal involvement. Patients with a prior history of ocular surgery or trauma; with a BCVA < 16/20; presenting with any systemic disease that might affect blood flow, such as diabetes mellitus or hypertension; with central serous chorioretinopathy, polypoidal choroidal vasculopathy, primary glaucoma, optic neuritis, age-related macular degeneration (AMD), retinal vein occlusion, choroidal melanoma and retinal vasculitis, uveitis, and epimacular membrane that could affect ocular circulation; or with moderate to high myopia (defined as a spherical equivalent refractive error (SERE) in phakic eyes <3.0 D), were excluded.

**Fig. 1:**
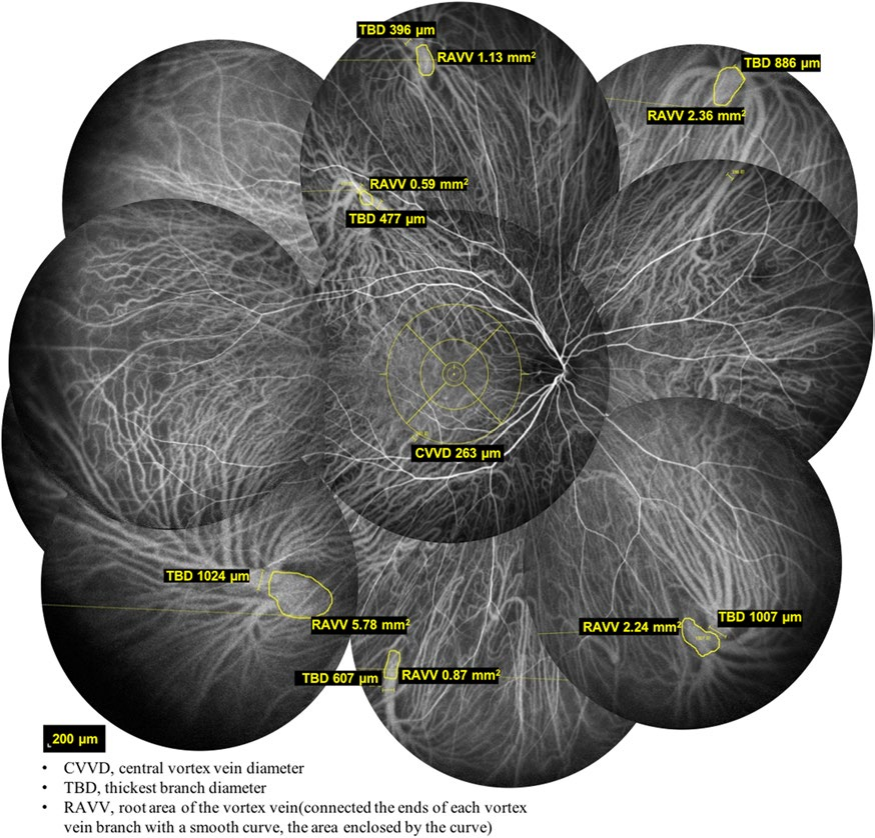
An ICGA fundus image of a 29-year-old man shows six independent vortex veins

**Fig. 2:**
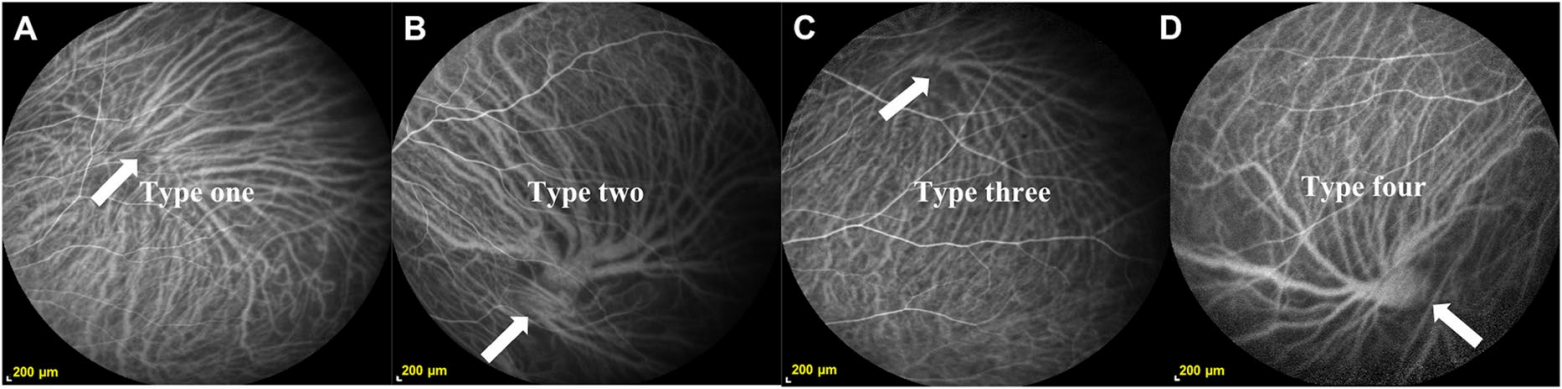
ICGA images of four types of vortex veins. In type one, the tributaries pass directly through the sclera instead of forming vortex veins (**A**). In type two, incomplete vortex veins arise where some tributaries pass through the sclera together with the vortex veins (**B**). In type three, complete vortex veins are seen where all tributaries join and then pass through the sclera (**C**). In type four, a complete system has formed, where all tributaries join the vortex veins that form an ampulla, which then passes through the sclera (**D**)

After intravenous injection of 5 mL of 25 mg indocyanine (SERB LAB, Paris, France), ICGA images were recorded with the retinal device (SPECTRALIS Diagnostic Imaging Platform; Heidelberg Engineering, Inc.). We selected images from the stage 10 min after dye injection for analysis. We recorded the patient’s sex, age, number of vortex vein roots (NVVR), location of vortex vein roots, and number of vortex vein branches (NVVB) and then used the sketching tool of the retinal device to mark the root area and the diameter of the thickest branch of each vortex vein. The center of the concentric circle is placed on the macula and the thickest branch of the vortex vein that intersects the outer ring with a diameter of 6 mm is selected, measured and stored as the central vortex vein diameter (CVVD). We connected the ends of each vortex vein branch with a smooth curve, and the area enclosed by the curve was defined as the root area of the vortex vein (RAVV). The width of the thickest first-order branch of the vortex vein was defined as the thickest branch diameter (TBD) (Fig. 1). Applying the calculation method below, the mean root area of the vortex vein (MRAVV) and the weighted mean of the thickest branch diameter (WMTBD) were obtained. The vortex veins were separated into four categories according to a previous method [13] (Fig. 2). In type one, the tributaries pass directly through the sclera instead of forming vortex veins (A). In type two, incomplete vortex veins arise where some tributaries pass through the sclera together with the vortex veins (B). In type three, complete vortex veins are seen where all tributaries join and then pass through the sclera (C). In type four, a complete system has formed, where all tributaries join the vortex veins that form an ampulla, which then passes through the sclera (D). We defined an anastomotic branch of the vortex vein as a branch of the vortex vein with a diameter of 125 μm and above that extends outward and then curves around to a vortex vein ampulla.

$$\begin{array}{c}\text{MRAVV}=\frac{\sum\text{RAVV}}{NVVR}\\\text{WMTBD}=\frac{\sum\left(\text{TBD}\ast\text{NVVB}\right)}{\sum\text{NVVB}}\end{array}$$

A total of 228 eyes underwent optical coherence tomography (OCT), and subfoveal choroidal thickness (SFCT) was measured using an OCT device (SPECTRALIS^®^ OCT; Heidelberg Engineering Inc.). All labeling work was performed separately by two experienced ophthalmologists, and the average measured by the two was taken as the final data.

### Analysis

One-way ANOVA was used to compare differences in the NVVR across age groups and quadrants and to compare the differences in the CVVD among the four age groups. When the ANOVA indicated a significant difference among multiple groups, Tukey’s test was used to perform pairwise comparisons. Pearson test was used to compare the correlation between age and MRAVV and WMTBD. Chi-square test was used to compare the proportions of type four vortex veins and the anastomotic branches of the vortex vein between people younger than 50 years old and those ≥50 years old. Correlation analysis was performed for age, NVVR, CVVD, MRAVV, WMTBD, and SFCT. Data are presented as the mean ± standard deviation. A value of *P* ≤ 0.05 was considered statistically significant. Statistical analysis was performed using SPSS for Windows version 17.0 (SPSS, Inc., Chicago, Illinois, USA).

## Results

This study included 228 eyes of 228 participants. Among the 228 participants, 117 (51.3%) were male, and 111 (48.7%) were female. The average age was 44.18±18.82 years (range 4–86 years), the average NVVR was 5.75±1.58 (range 2–10), the average CVVD was 223.81±69.10 μm (range 107–531 μm), the MRAVV was 1.69±0.63 mm^2^ (range 0.49–4.36 mm^2^) and the WMTBD was 481.50±104.08 μm (range 233–859 μm).

**Fig. 3:**
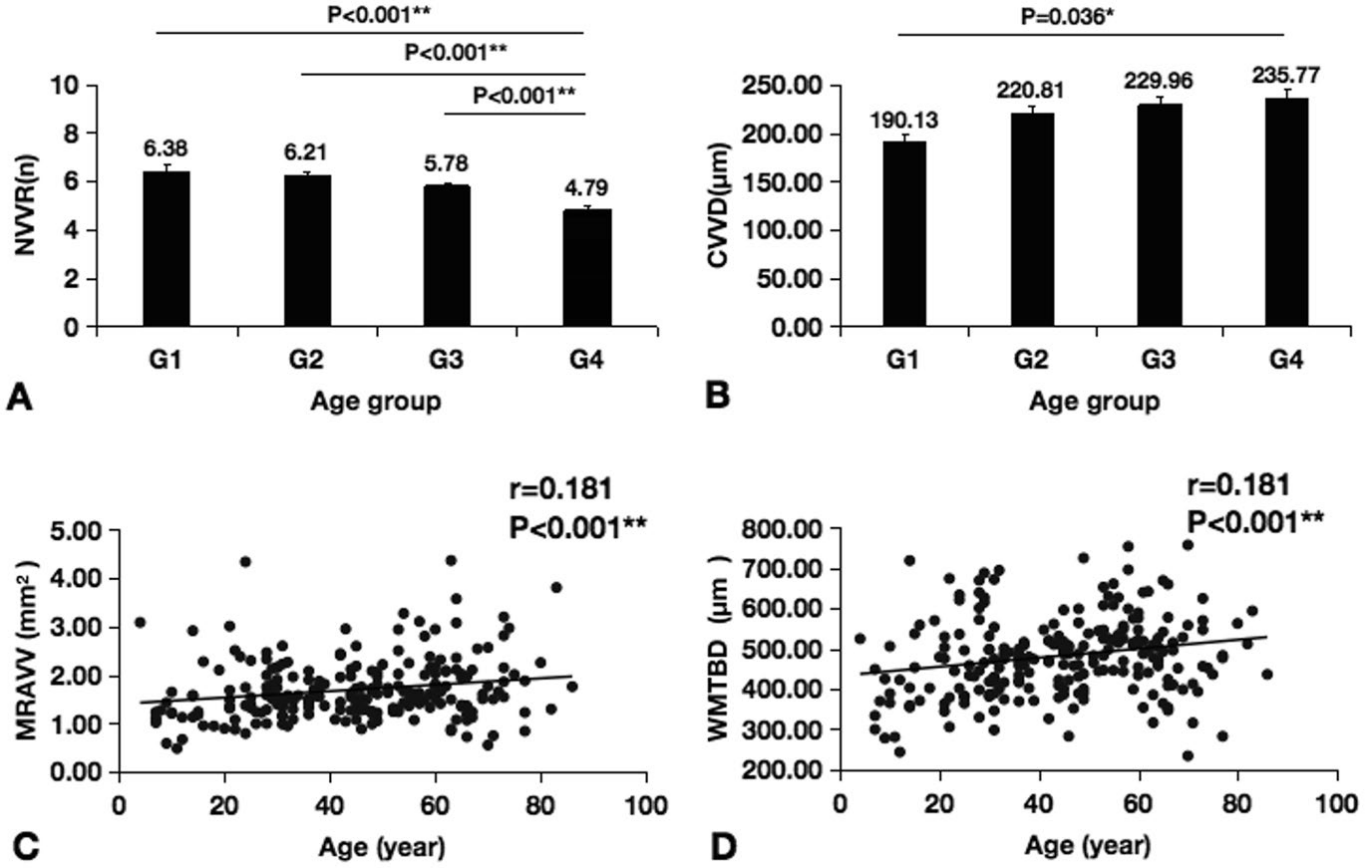
Relations between age and NVVR, CVVD, MRAVV, and WMTBD. The NVVR was not identical across age groups, and the NVVR in the over 60 years age group was significantly lower than that in the other age groups (*P* < 0.05) (**A**). The CVVD was different in the four age groups and showed an increasing trend with age (**B**). There were significant differences between the G1 group and the G3 group and between the G1 group and the G4 group (*P* < 0.05). With increasing age, the MRAVV and WMTBD of all age groups also increased (*P* < 0.001) (**C–D**). NVVR, number of vortex vein roots; CVVD, central vortex vein diameter; MRAVV, mean root area of the vortex vein; WMTBD, weighted mean of the thickest branch diameter. (*P < 0.05, **P < 0.001)

**Fig. 4:**
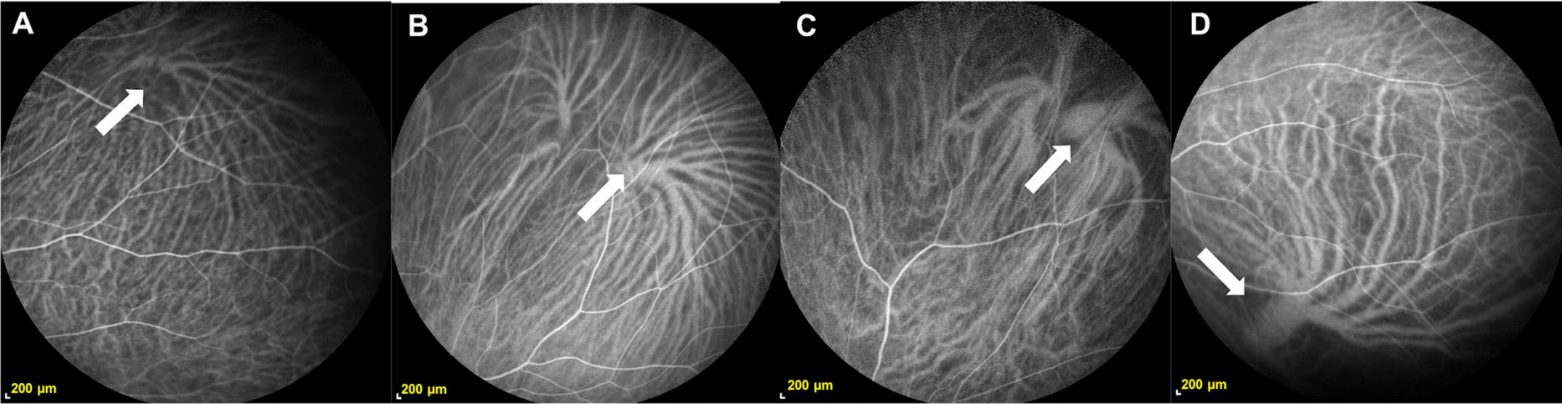
ICGA images of the vortex veins in different age groups. The vortex veins tend to expand with age. Image from an 8-year-old boy (**A**). Image from a 29-year-old male (**B**). Image from a 46-year-old female (**C**). Image from a 77-year-old male (**D**)

Participants were divided into 4 groups according to age. There are 24 participants (mean age, 12.13 ± 4.17 years; range, 4–20 years) in G1, 73 participants (mean age, 30.14 ± 5.18 years; range, 21–40 years) in G2, 79 participants (mean age, 50.85 ± 5.89 years; range, 41–60 years) in G3, and 52 participants (mean age, 68.56 ± 6.14 years; range 61–86 years) in G4. There was no significant difference in gender distribution among the four groups. The differences in the NVVR, CVVD, MRAVV, and WMTBD among each age group were compared, and the results are shown in Fig. 3. The NVVR was not identical across age groups, and the NVVR in the over 60 years age group was significantly lower than that in the other age groups (*P* < 0.05) (Fig. 3A). CVVD was different in the four age groups and showed an increasing trend with age; additionally, the differences between the G1 group and the G4 group were significant (*P* < 0.05) (Fig. 3B). With increasing age, the MRAVV and WMTBD of all age groups also increased (*P* < 0.001) (Fig. 3C and D). With increasing age, the CVVD, MRAVV, and WMTBD of all age groups all increased, indicating that the vortex vein tended to dilate over time (Fig. 4).

We divided the fundus into four quadrants, superior-temporal, inferior-temporal, superior-nasal, and inferior-nasal, and counted the number and types of vortex veins in each quadrant. The results are shown in Table 1. The results showed that the number of vortex veins was unevenly distributed among the quadrants (*P* < 0.001) (Table 1); specifically, the number of vortex veins on the temporal side was greater than that on the nasal side (*P* < 0.05) and the number of vortex veins between the two temporal regions and between the two nasal regions was basically the same (Table 1).

**Table 1:** The number of vortex veins in each quadran

	Mean ± SD	Range	*F*	*P*
Superior-temporal	1.56 ± 0.66	0–4	17.032	<0.001**
Inferior-temporal	1.61 ± 0.65	0–3		
Superior-nasal	1.35 ± 0.63	0–3		
Inferior-nasal	1.24 ± 0.61	0–4		

**P<0.001

We classified all vortex veins in 228 eyes and found that type III (complete) had the largest proportion, similar to that of type I (vortex vein absent), and the proportions of type II (incomplete) and type IV (complete with ampulla) vortex veins were also similar. We compared the proportions of type four vortex veins and anastomotic branches in people under 50 years old and those over 50 years old and found significantly increased values in the older group (*P* < 0.05).

Correlations of SFCT with age, NVVR, CVVD, MRAVV, and WMTBD were investigated retrospectively with OCT data collected from the 228 eyes. The average SFCT was 279.45 ± 85.02 μm (range 114–510 μm); it was not significantly associated with WMTBD (*P* > 0.05) but was significantly correlated with age, NVVR, CVVD, and MRAVV (*P* < 0.05).

## Discussion

Aging is associated with neurological and vascular degeneration, which may lead to anatomical and functional changes. This is the first study to reveal age-related alterations in vortex veins on indocyanine green angiography in a healthy population. Our study found that the NVVR in the age group over 60 is significantly lower than that in other age groups, and the CVVD, MRAVV, and WMTBD of all age groups increased with increasing age. This suggests that aging may lead to partial vortex vein occlusion, scleral sclerosis leads to increased pressure in the vortex veins, and residual vortex veins exhibit compensatory dilation, manifesting as filling and dilation of vortex vein roots and branch vessels. Additionally, anastomotic branches appear between initially separate vortex veins.

The NVVR was different in the four age groups and decreased with age. The NVVR in the age group over 60 years old was significantly lower than that in other age groups. We speculate that elderly individuals may demonstrate small vortex vein occlusion, which is a normal physiological phenomenon. Similar to the results of the present study, Chen et al. [14] found that the number of intracranial vascular branches decreased with increasing age by using iCafe. Hayreh et al. showed that experimental occlusion of less than three vortex veins would not cause any deleterious effect on retinal or choroidal tissue, which might be due to choroidal vascular remodeling and opening of new drainage channels [15–17]. This may explain why NVVR is reduced but no disease is present in most elderly people.

The CVVD and RAVV continue to expand with age, while the WMTBD decreases after peaking at 41–60 years (G3). Similarly, Lin et al. [11] found that retinal tissue perfusion decreased with age after 35 years and was significantly reduced in subjects aged more than 65 years old. From infancy to adulthood, the body undergoes continuous growth and development. During this process, the branches of the vortex veins gradually thicken, and the ampulla enlarges, both of which are growth phenomena. Yu et al. [18] found an increase in the area of vortex vein endothelial cells associated with aging, suggesting that aging may lead to changes in the vortex veins. Studies have shown that in human sclera, the number of elastin fibers decreases after 20 years [19], decorin and biglycan levels decrease after 40 years [20], and the cross-sectional area of scleral collagen fibrils increases with age [21], which may be a determinant of scleral sclerosis [22]. Aging-induced scleral sclerosis may increase the flow resistance of the vortex veins in the intrascleral channel [18–23]. We speculate that due to scleral sclerosis, the congestion of the vortex veins is aggravated, and the area of the roots of the vortex veins continues to expand. This may also be the reason why polypoid choroidal vascular disease is more common in elderly individuals.

The proportion of type four vortex veins and anastomotic branches in participants over 50 years of age was significantly higher than that of participants under 50 years of age. We classified all vortex veins in 228 eyes and found that type three had the largest proportion, similar to that of type 1, and the proportions of type 2 and type 4 vortex veins were also similar. That is, most of the vortex vein tributaries converged after passing through the sclera [13]. Unsurprisingly, the most noticeable feature of type four is the complete formation of the vortex venous system, in which all the tributaries converge and form the ampulla, which then penetrates the sclera. Based on previous findings, we divided the participants into two groups, those over 50 years old and those under 50 years old, and found that the proportions of type four vortex veins and anastomotic branches were significantly higher in the older age group. Hayreh et al. demonstrated that there were no anastomoses between two vortex veins under physiological conditions [24]. This suggests that the shape of the vortex veins is not static, but with age, the blood vessels thicken, the ampulla enlarges, and anastomotic branches appear between the vortex veins. Chen et al. [25] experimentally occluded two temporal vortex veins in monkeys and found increased supratemporal choroidal thickness after 1 day and 4 weeks of occlusion and increased supranasal and infranasal choroidal thickness after 12 weeks of occlusion. Hidetaka Matsumoto et al. [26] ligated superotemporal and inferior temporal vortex veins in monkey eyes and observed dilatation of superotemporal and inferior temporal vortex veins and anastomosis of intervortex veins, and B-type OCT images showed choroidal thickening. This may be a potential compensatory mechanism by which aging leads to occlusion of some small vortex veins, compensatory expansion of residual vortex veins and anastomosis. The specific manifestations are enlargement of the ampulla and thickening of the vortex veins.

The SFCT was significantly correlated with age, NVVR, CVVD, and MRAVV. In our study, SFCT was negatively correlated with age, positively correlated with NVVR, and negatively correlated with MRAVV and CVVD. With age, the NVVR decreased, and the MRAVV and CVVD increased. That is, aging leads to partial vortex vein occlusion, and residual vortex vein dilation can only partially compensate, resulting in a decrease in choroidal thickness. Matsumoto et al. [27] also found that central choroidal thickness (CCT) was correlated with the mean diameter of the vortex veins. Apparently, the choroidal vessels are filled better, and the subfoveal choroid becomes thicker, as shown on OCT. The SFCT measured by OCT was correlated with the NVVR, CVVD, MRAVV on ICGA. This study demonstrated that SFCT was not significantly associated with WMTBD (*P* > 0.05) but was significantly correlated with CVVD (*P* < 0.05). This may be because the branch of the vortex veins measured by WMTBD is located at the equator, while the branch of the vortex vein measured by CVVD is located at the posterior pole, which is closer to the fovea, so it has a stronger correlation with the SFCT.

### Limitations

This study is a cross-sectional study, and it would be desirable to conduct a longitudinal study in the future to observe the changes in vortex veins with age in the long term. Due to ethical reasons, we cannot collect a large number of normal people’s ICGA pictures. We selected the opposite eye with unilateral eye disease and no choroid involvement. Although this can be used for preliminary research, it cannot be guaranteed that the value is completely consistent with that of the normal population.

## Conclusions

This study described various parameters, such as the number, branch diameter, root area, location, type and anastomosis of vortex veins in each age group, and proposed the central vortex vein diameter, the mean root area of vortex veins and the weighted mean of the thickest branch diameter of vortex veins as parameters for quantitative evaluation of these vessels. Ultimately, this study sheds light on the relationship between aging and vortex varicose veins, finding that aging is associated with partial vortex vein occlusion and residual vortex vein dilation. The correlation between the subfoveal choroidal thickness and the number of vortex veins, posterior vortex vein diameter, and mean root area of the vortex vein was verified, which further confirmed the significance of the parameters we proposed. At present, there are relatively few studies on vortex veins, and this study provides a basis for further exploration of the relationship between these vessels and age.

## Data Availability

Data are available upon reasonable request.

## Abbreviations

NVVR: number of vortex vein roots
NVVB: number of vortex vein branches
CVVD: central vortex vein diameter
RAVV: root area of the vortex vein
MRAVV: mean root area of the vortex vein
TBD: thickest branch diameter
WMTBD: weighted mean of the thickest branch diameter
ICGA: indocyanine green angiography
CSC: central serous chorioretinopathy
OCTA: optical coherence tomography angiography
RTP: retinal tissue perfusion
VVD: volumetric vessel density
SVP: superficial vascular plexus
PCV: polypoidal choroidal vasculopathy
AMD: age-related macular degeneration
SFCT: subfoveal choroidal thickness
CCT: central choroidal thickness

## Declarations

### Patient and public involvement

Patients and/or the public were not involved in the design, conduct, reporting, or dissemination plans of this research.

### Provenance and peer review

Not commissioned; externally peer reviewed.
